# Research progress and hotspot analysis of rhizosphere microorganisms based on bibliometrics from 2012 to 2021

**DOI:** 10.3389/fmicb.2023.1085387

**Published:** 2023-02-23

**Authors:** Shangsheng Sun, Ruipeng Xue, Mengyue Liu, Liqing Wang, Wei Zhang

**Affiliations:** ^1^Engineering Center for Environmental DNA Technology and Aquatic Ecological Health Assessment, Shanghai Ocean University, Shanghai, China; ^2^Centre for Research on Environmental Ecology and Fish Nutrient of the Ministry of Agriculture, Shanghai Ocean University, Shanghai, China

**Keywords:** rhizosphere microorganisms, bibliometric analysis, research hotspot, thematic evolution, visualized analysis

## Abstract

Rhizosphere microorganisms are important organisms for plant growth promotion and bio-control. To understand the research hot topics and frontier trends of rhizosphere microorganisms comprehensively and systematically, we collected 6,056 publications on rhizosphere microorganisms from Web of Science and performed a bibliometric analysis by CiteSpace 6.1.3 and R 5.3.1. The results showed that the total number of references issued in this field has been on the rise in the past decades. China, India, and Pakistan are the top three countries in terms of the number of articles issued, while Germany, the United States, and Spain were the countries with the highest number of co-published papers with other countries. The core research content in this field were the bio-control, bacterial community, ACC deaminase, phytoremediation, induced systematic resistance, and plant growth promotion. Seeding growth, *Bacillus velezensis*, plant-growth, and biological-control were currently and may be the highlights in the field of rhizosphere microorganisms research for a long time in the future. The above study results quantitatively, objectively, and scientifically described the research status and research focus of rhizosphere microorganisms from 2012 to 2021 from the perspective of referred papers, with a view to promoting in-depth research in this field and providing reference information for scholars in related fields to refine research trends and scientific issues.

## 1. Introduction

The concept of the “rhizosphere” was first proposed in 1904 by German microbiologist Lorenz Hiltner while illustrating the relationship between plants and bacteria, and was defined as the soil around the roots of plants affected by root growth (Fields and Friman, 2022). Rhizosphere microorganisms are one of the most complex microbial communities on the earth with a large number and rich diversity (Mendes et al., 2013), which mainly include bacteria and fungi (Gu et al., 2021). Like the second set of plant genomes, they play a crucial role in plant growth and development, nutrient acquisition, pest prevention, and yield improvement (Mendes et al., 2011). Plants affect rhizosphere microbial community structure by secreting root exudates, and even there is a certain degree of dependence and specificity between plants and rhizosphere microorganisms (Paterson et al., 2007; Nakayasu et al., 2021). On the contrary, the change in rhizosphere microbial community structure will also affect the release of plant root exudates, the material circulation, the energy flow of the soil system, thus affecting the growth, development, and diversity of plant communities (Eisenhauer et al., 2012; Wu et al., 2022). In the past decades, scholars have done a lot of research on rhizosphere microorganisms (Abdullaeva et al., 2021; Ye et al., 2021). For example, Yang et al. (2022) studied the alleviative effect of rhizosphere microorganisms on plant heavy metal stress and its mechanism, while Kui et al. (2021) studied a comparative analysis on the structure and function of rhizosphere microbiome on *Panax notoginseng*. To sum up, predecessors have done a lot of research, but the research hotspots and development trends in this field are still unclear.

Bibliometric analysis is a method to analyze the research characteristics and change trends of literature by using a variety of mathematical and statistical techniques (Liu et al., 2019). Using this method, we can quantitatively calculate the literature overview, cooperative relationship, research hotspots, and scientific research development trends of a certain research field (Guan et al., 2021). Bibliometric analysis has been widely used in academic research, especially in the field of microorganism. For example, Yuan et al. (2021) and the others summarized the research focus and trends of human intestinal microorganisms from 2010 to 2021 through bibliometrics; Ekundayo et al. (2021) published a global bibliometric meta-analytic assessment of research trends on microbial chlorine resistance in drinking water/water treatment systems in 2021. CiteSpace, a visual analysis tool, which is used to capture trends and patterns in the scientific literature (Chen et al., 2014). CiteSpace can be used to explore the scientific domains in a research community from the perspective of problem solvers and discoverers (Guan et al., 2021).

In this study, we conducted a descriptive analysis of published research on rhizosphere microorganisms, analyzed the co-occurrence of keywords in published articles, and drew a theme evolution map to explain the changes in scientific themes. The aim of this work is to summarize the research of rhizosphere microorganisms and predict the hot research directions in the future.

## 2. Methods

### 2.1. Data source and search strategy

Literature retrieval was performed online through the Science Citation Index Expanded (SCI-E) of the Web of Science Core Collection (WoSCC, Clarivate Analytics) on September 18, 2022. The Web of Science database is considered a preferred data source for bibliometric analysis due to the comprehensive information and multi-disciplinary data of literature provided (Falagas et al., 2008; Archambault et al., 2009). To retrieve literature comprehensively and accurately on rhizosphere microorganisms, different search terms and retrieval strategies were assembled in this study. Finally, the optimal search items were set as follows: TS = ((“Rhizosphere microorganism*”) OR (“Rhizosphere microbe*”) OR (“Rhizosphere bacteria”) OR (PGPR) OR (PGPB) OR (PGPF) OR (Rhizobacteria) OR (“Rhizosphere fungi”) OR (“Rhizosphere fungus”)). The data range was set from January 1, 2012, to December 31, 2021, to collect all relevant publications. It is worth noting that as the Web of Science data network is constantly updated, the results may vary depending on the exact retrieval date.

### 2.2. Publication collection and exclusion criteria

**Figure 1:**
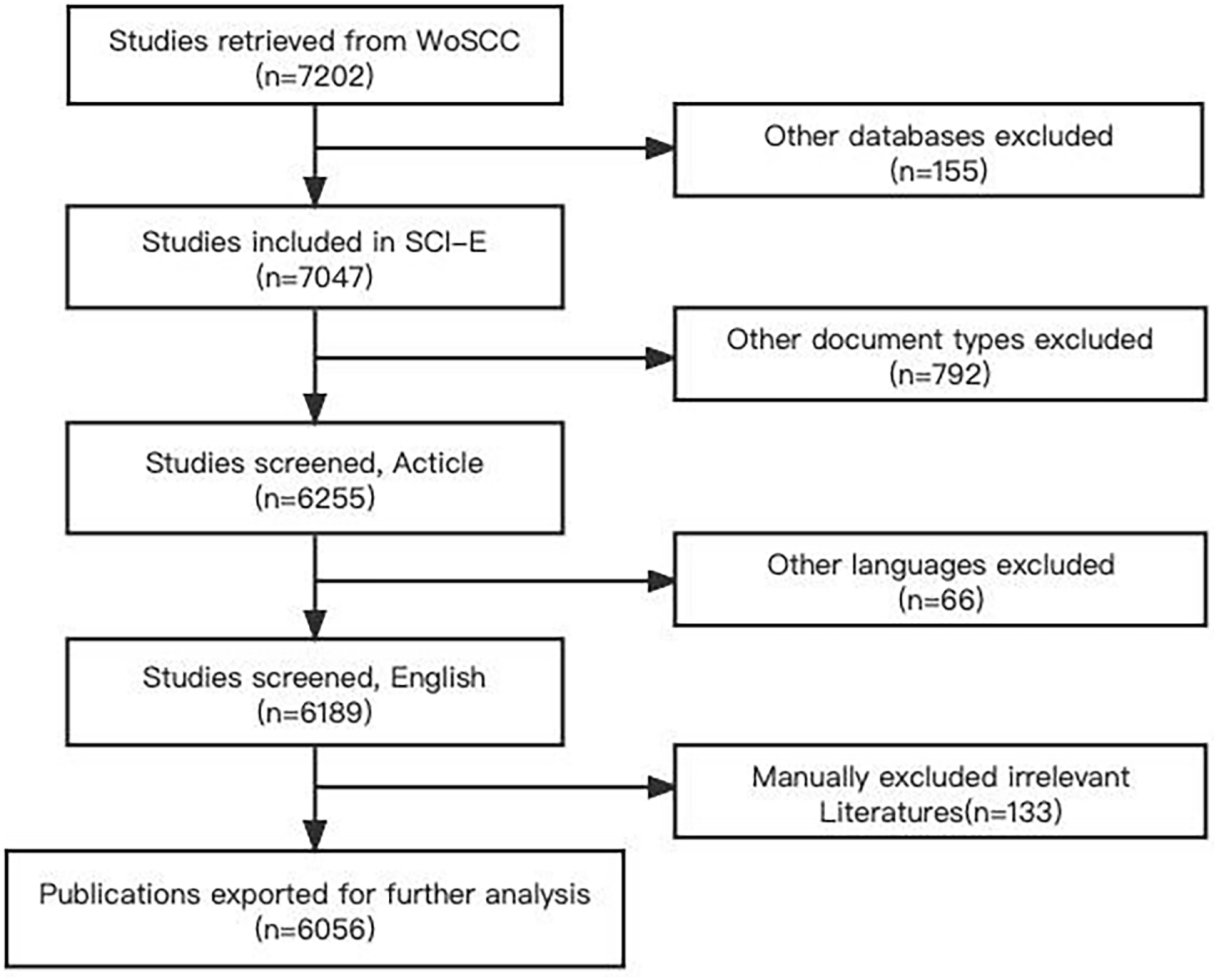
The flowchart of retrieving publications. WoSCC: the Web of Science Core Collection; SCI-E: the Science Citation Index Expanded.

To ensure the reliability of the research conclusions, this study collected literature from peer-reviewed English journals to summarize research perspectives worldwide. It is important to note that some acquired articles not in line with the purpose of this paper were excluded manually in the last step of data collection. For instance, some studies discussed the relationship between plant and phyllospheric microorganisms. Eventually, a total of 6,056 pieces of literature that met all criteria were downloaded from WoSCC. These articles represented almost all of the high-quality experimental studies on the rhizosphere microorganisms around the world from 2012 to 2021. The variation of these literatures could powerfully indicate the development tendency of relevant research. Therefore, we focused on these publications for further analysis and evaluation. Figure 1 shows the flowchart of these literatures retrieved in this work (Sun et al., 2020).

### 2.3. Data visualization

This article mainly used a Java-based visualization tool, CiteSpace 6.1.3, to visualize the results of bibliometric and content analysis. CiteSpace, a scientific metrology software developed by Chen (2006), can quantitatively calculate and visualize multiple co-occurrence networks, including core countries/regions, institutions, authors, and references, to uncover potential connections and variances. The time distribution characteristics of keywords can also be shown by CiteSpace to find research focus and future directions within a given research domain (Zhu and Hua, 2017). In addition, we also use the function “biblionshirny” in the “bibliometrix” package (Aria and Cuccurullo, 2017) in R 3.5.1 to do the thematic evolution analysis.

## 3. Results and discussion

### 3.1. Bibliometric and cooperation network analysis

#### 3.1.1. Annual publication number

**Figure 2:**
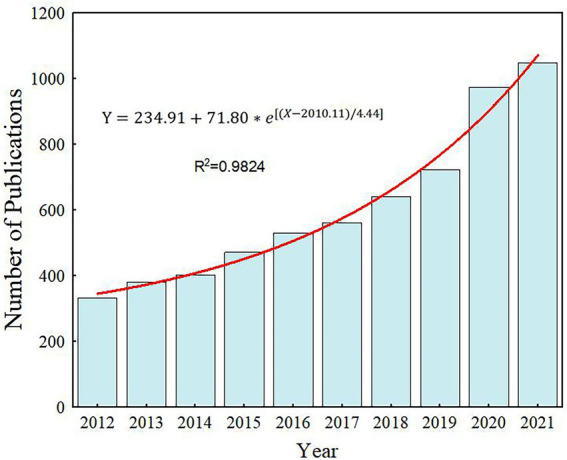
The distribution of publications on rhizosphere microorganisms from 2012 to 2021.

The change in annual publication counts is a crucial index to estimate the advance of the research field, which is advantageous for the prediction of the development trend (Sun et al., 2020). Figure 2 clearly showed the distribution of publications on rhizosphere microorganisms in different periods. It could be seen that the number of articles published in this field was increasing year by year on the whole, and the annual growth rate was 13.66% after calculation. So could be inferred that this field has attracted much attention in the past few years (Cheng et al., 2020). The development of a scientific research field generally goes through four stages (Sun et al., 2020): (1) the initial stage, in certain famous scientists deliver new research fields or directions; (2) the growth stage, scientists are pyramidally attracted to this research direction and develop an increasing number of discussion topics; (3) the stable development stage, new knowledge is integrated to form a specific research context; (4) the recession stage, the number of new publications decrease. Obviously, the research on Rhizosphere microorganisms is still in the growth stage (Li et al., 2022).

#### 3.1.2. Publication contribution analysis

The 10 countries/regions, institutions, authors, and journals that published the most rhizosphere microorganisms’ studies were listed in Table 1. As shown in Table 1, China was the country that made the highest contribution, with 1,231 publications (20.33% of the total). India and Pakistan ranked second and third, contributing 1,096 (18.10%) and 618 (10.20%) publications, respectively. Among the institutions, the Chinese Academy of Sciences was the greatest contributor, with 160 (2.64%) publications. The second and third largest institutional contributors were the University of Agriculture, Faisalabad (144, 2.37%) and Nanjing Agricultural University (137, 2.26%). Scholars with many high-quality publications often dominate the development trends of the research, and identifying influential scholars can indicate who led the research (Yang et al., 2020). The most active author in the field of rhizosphere microorganisms was Wang Y. (with 102 publications). Li Y, Zhang Y, and Hkan M were similarly prolific, each publishing almost 90 studies. These publications appeared mainly in mainstream journals of Ecology and Botany science, including *Frontiers in Microbiology* (3.73%), *Frontiers in Plant Science* (2.36%), and *Plant and Soil* (2.03%).

**Table 1:** The 10 countries/regions, institutions, authors, and journals with the most rhizosphere microorganism publications from 2012 to 2021.

Rank	Country	Numbers	Institution	Numbers	Author	Numbers	Journ source	Numbers
1	China	1,231	Chinese Academy of Sciences	160	Wang Y	102	*Frontiers in Microbiology*	226
2	India	1,096	University of Agriculture, Faisalabad	144	Li Y	98	*Frontiers in Plant Science*	143
3	Pakistan	618	Nanjing Agricultural University	137	Zhang Y	83	*Plant and Soil*	123
4	the United States	564	Chinese Academy of Agricultural Sciences	104	Hkan M	83	*Applied Soil Ecology*	121
5	Brazil	306	King Saud University	81	Li X	67	*Microbiological Research*	121
6	South Korea	294	Quaid-i-Azam University	79	Bano A	65	*Plos One*	113
7	Iran	291	Kyungpook National University	72	Zhang J	65	*Scientific Reports*	104
8	Spain	282	Bahauddin Zakariya University	72	Liu Y	61	*Biological Control*	93
9	Germany	269	University of Chinese Academy of Sciences	64	Wang J	57	*Environmental Science and Pollution Research*	87
10	Canada	201	University of the Punjab	62	Khan A	51	*Agronomy-Basel*	84

#### 3.1.3. Scientific collaboration analysis

**Figure 3:**
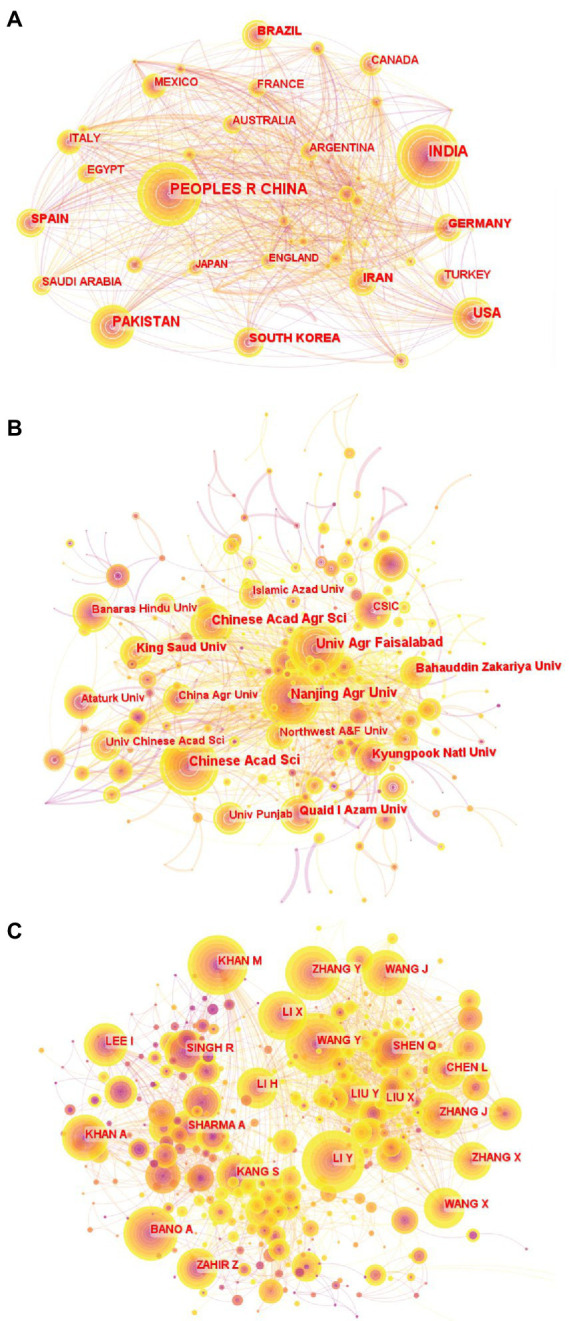
Cooperation network of major countries/regions engaged in rhizosphere microorganisms research **(A)**. Cooperative network of major institutions engaged in rhizosphere microorganisms research **(B)**. Cooperative network of leading authors engaged in rhizosphere microorganisms research **(C)**.

According to Figure 3A, different countries/regions had frequent cooperation in rhizosphere microorganisms (Liao et al., 2020). In this study, the degree of international cooperation was assessed by the number of articles jointly published by the two countries/regions. The size of a node indicated the number of published articles, while a larger node indicated that more articles have been published (Zhang Y. et al., 2020). The width of the connecting line between the two countries indicated their connection. The wider the line was, the more articles were published by the two countries through cooperation (Li et al., 2022). Although China, India, and Pakistan were the top three countries in terms of the number of articles issued, the number of cooperative articles issued with other countries was not in this order. Germany, the United States, and Spain ranked among the top three countries in terms of the number of articles issued in cooperation with other countries, and their center rates were 0.25, 0.23, and 0.16, respectively. Therefore, we can draw a conclusion that the number of papers published did not mean that there was more cooperation with other countries (He et al., 2022). Academic level and geographical location maybe are the primary considerations for researchers to seek international cooperation in this field (Zeng et al., 2022).

Analyzing the distribution of research institutions can help to understand the degree of support and recognition of the academic community in this field, which is conducive to cooperation between institutions (Zhang Y. et al., 2020). Figure 3B showed that the three organizations with the largest number of documents were Chinese Academy of Sciences, University of Agriculture, Faisalabad and Nanjing Agricultural University. Among them, Nanjing Agricultural University and King Saud University had the largest number of cooperation with other institutions, and their centrality were both 0.23, which may be due to the establishment of a research center responsible for cooperative research in the field of rhizosphere microorganisms (Liu et al., 2019). In addition, Chinese Academy of Sciences (centrality 0.13) and University of Agriculture, Faisalabad (centrality 0.11) also showed extensive cooperation. This information may help global scholars find influential research institutions and potential collaborators to establish academic exchanges in this field (Liu et al., 2022). However, from a global perspective, most scientific research institutions still focused on independent research, and the internal cooperation of each consortium was much higher than the external cooperation (Zeng et al., 2022).

The team effect of academic research can be explored by closely cooperating scholars (Ma et al., 2020). Wang Y, Li Y and Zhang Y were the three authors with the largest number of published papers (Figure 3C). However, from the number of connections, Khan M (Centrality 0.35), Singh R (Centrality 0.21), and Kumar A (Centrality 0.21) were the main authors who had more contacts with other researchers in the cooperation network, which showed that they have a strong influence in the field of rhizosphere microorganisms research (Xiao et al., 2022). From a global perspective, most of the nodes in the figure were independent and decentralized, and most researchers focused on small-scale independent research, which indicated that most rhizosphere microorganisms researchers were widely distributed and independent (He et al., 2022).

### 3.2. Keywords analysis

#### 3.2.1. Keywords co-occurrence network and cluster analysis

**Figure 4:**
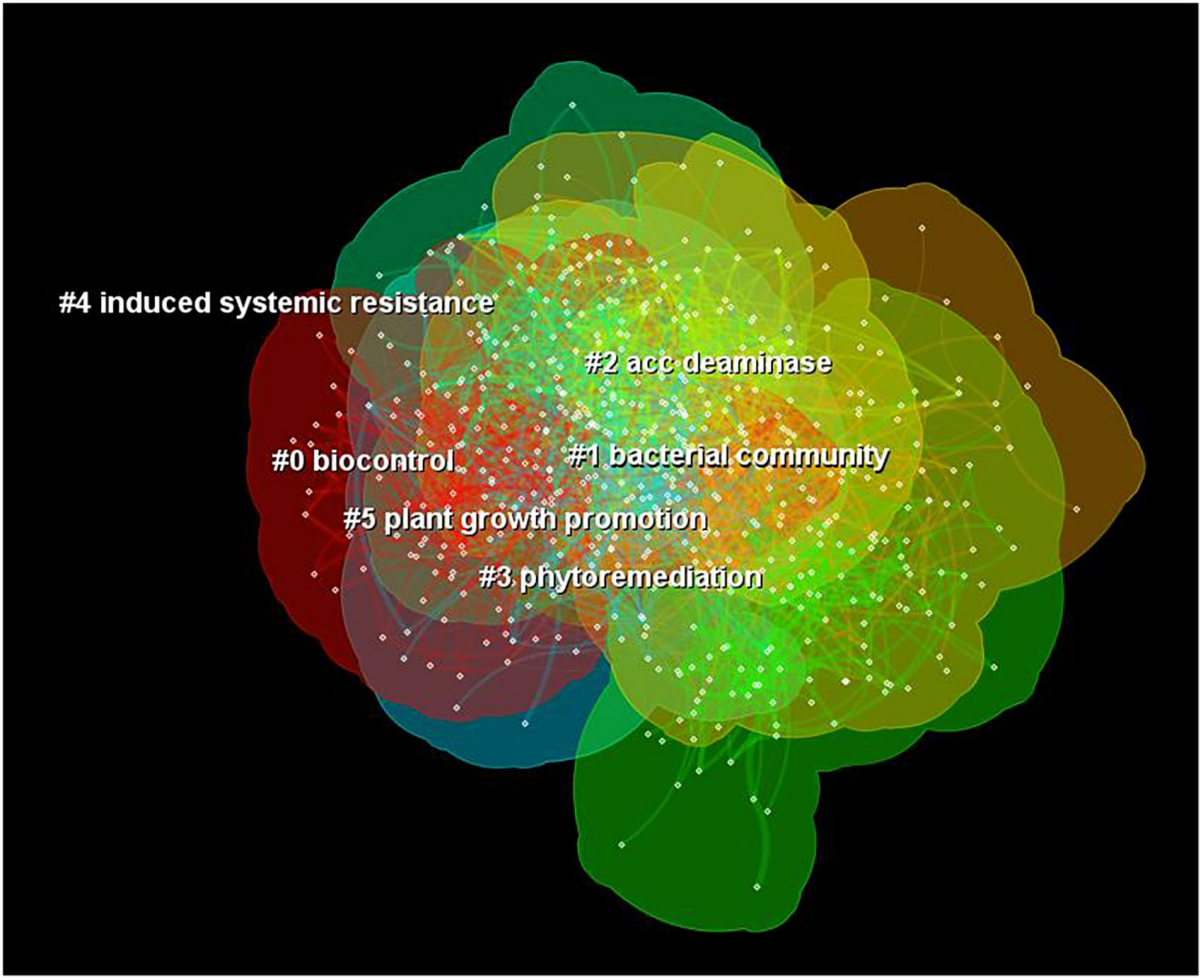
Cluster map of co-occurring keywords in rhizosphere microorganism field published from 2012 to 2021.

The keywords have highly refined and summarized the core content of the article, and their frequency can reflect the research direction and content of a certain field (Zhang Z. et al., 2020). Through the analysis of the keywords co-occurrence networks, the development trend of a certain field can be identified, and the research status in this field can be grasped (Yan et al., 2021). After analyzing the co-occurrence graph of a single keyword, we used the log-likelihood ratio (LLR) algorithm to draw the co-occurrence cluster graph of keywords. Co-occurring clusters were arranged according to their size. A total of 6 co-occurrence clusters were obtained (Figure 4), including “bio-control” (#0), “bacterial community” (#1), “ACC deaminase” (#2), “phytoremediation” (#3), “induced systemic resistance” (#4), and “plant growth promotion” (#5). Each cluster was discussed in detail below to better describe the hot topics in the rhizosphere microorganisms’ field during the study.

#### 3.2.2. Cluster #0: “Bio-control”

In recent years, bio-control has gradually attracted rising attention due to its advantages of non-pollution, high efficiency, and low cost (Shen et al., 2013). Bio-control generally adopts the method of applying biological organic fertilizer. This method can not only reduce the use cost of pesticides and fertilizers, and the efficiency of nutrients in the soil, but also improve the diversity of soil microorganisms (Moustafa and Taha, 2021), which is the best control measure to reduce the occurrence of plant diseases (Mousa et al., 2021). In respect of biological control, rhizosphere microorganisms play an important role in the healthy growth of plants (Wei et al., 2022).

#### 3.2.3. Cluster #1: “Bacterial community”

Studies showed that the abundance and diversity of rhizosphere bacterial community was important indicators of rhizosphere health (Ren et al., 2020). Because bacteria can form symbiotic networks in the rhizosphere, it can not only express a variety of related molecular patterns in rhizosphere symbiotic microorganisms (Wang W. et al., 2022), but also these stable symbiotic structures of bacterial communities can trigger plant defense mediated by these pattern when the environment changes, so that plants will react quickly (Liu et al., 2020), and then induce plant individuals or their descendants to enhance the resistance (Berendsen et al., 2018). Furthermore, the rhizosphere microbial community also has a profound significance on plant growth, such as increasing nutrient absorption, improving root structure, and inhibiting diseases (Van der Voort et al., 2016; Bakker et al., 2018).

#### 3.2.4. Cluster #2: “ACC deaminase”

Ethylene can inhibit the elongation and growth of stems, promote the thickening of stems or roots, and promote the lateral growth of stems, which is called the triple reaction of ethylene (Gupta and Pandey, 2019). 1-Aminocyclopropane-1-carboxylic acid (ACC) is the direct precursor of ethylene biosynthesis, and ACC deaminase can decompose ACC into α-Butyruvic acid and ammonia, thus reducing the ACC content in plants, indirectly alleviating the triple reaction of ethylene to plants (Gupta and Pandey, 2019). A large number of studies have shown that applying ACC deaminase-producing strains can effectively reduce the ethylene content produced by plants under abiotic stress conditions such as high salinity (Goel et al., 2019; Gupta et al., 2020), drought (Chandra et al., 2018), and waterlogging (Glick and Nascimento, 2021). Therefore, the use of plant rhizosphere microorganisms that produce ACC deaminase can promote the growth of plants under stress conditions (Moon and Ali, 2022).

#### 3.2.5. Cluster #3: “Phytoremediation”

Specific plant rhizosphere microorganisms can promote plant growth and adaptability, protect plants from pathogens, improve plant tolerance to heavy metals, and improve plant nutrient absorption and its absorption and transport of heavy metals (Manoj et al., 2020). This is achieved by producing various compounds, such as organic acids, iron carriers, antibiotics, enzymes, and plant hormones (Ma et al., 2011). Arbuscular mycorrhizal fungi (AMF) is an important rhizosphere microorganism to help plants carry out phytoremediation (Zhang et al., 2019). The presence of AMF in the rhizosphere increases the absorption surface area of plant roots through extensive mycelial networks, thereby improving the absorption of water and nutrients and the bioavailability of heavy metals (Song et al., 2021). AMF can also produce phytohormones that promote plant growth and help plant repair their environment (Chen et al., 2019). At the same time, plants, bacteria, and fungi have been shown to possess pesticide-degrading capacities, which can be applied in the successful remediation of contaminated fields and water (Eevers et al., 2017). As for the environmental pollution caused by the discharge of dyes, research shows that phytoremediation applies to the treatment of dye pollutants from various sources (Gayathiri et al., 2022).

#### 3.2.6. Cluster #4: “Induced systemic resistance”

Some growth-promoting bacteria can mediate induced systemic resistance (ISR) in plants, and produce resistance to a variety of fungi, bacterial, and viral diseases, and even to some pests and nematodes (Pieterse et al., 2014; Köhl et al., 2019; Mhatre et al., 2019). ISR does not need to directly activate plant defense response, but it can quickly and strongly initiate immunity when pathogens invade (Dreischhoff et al., 2020). For instance, soybean treated with the bacterium *Bacillus* sp. CHEP5 remarkably stimulated the intrinsic resistance of the plant to the fungus *Cercospora sojina* Hara, an agent that causes frogeye leaf spot (FLS) disease in soybean plants (Enebe and Babalola, 2019). It equally empowered the plant to express defense genes responsible for jasmonic acid synthesis (Tonelli and Fabra, 2014). Compared with the efficacy of chemical control and antagonistic bacteria, induced systemic resistance is generally pollution-free, broad-spectrum, systematic, and non-specific, and will not cause resistance to pathogenic microorganisms (Dreischhoff et al., 2020; Nishad et al., 2020).

#### 3.2.7. Cluster #5: “Plant growth promotion”

Most rhizosphere microorganisms have the physiological functions of dissolving phosphorus, dissolving potassium, and fixing nitrogen (Pandey et al., 2020). They release soluble potassium and phosphorus for crops to absorb and use in some ways. They can also meet the needs of crops for nitrogen by converting N_2_ in the air into NH_3_, so as to improve the absorption and utilization of nutrients by crops，which could promote the growth and development of crops (Fields and Friman, 2022; Wang S. et al., 2022). Certain specific rhizosphere microorganisms such as *Pseudomonas putida* CO1 can secrete some plant growth-regulating substances, such as plant hormones, enzymes, etc. These hormones and enzymes play a role in promoting the growth and development of plants by regulating their growth metabolism (Pandey et al., 2020; Bhojiya et al., 2022). Iron exists in nature in a form that is difficult to be absorbed and utilized by plants. Some microorganisms can produce iron carriers, which can synthesize chelates with Fe specifically to increase the content of effective iron, thereby promoting the absorption and utilization of iron by plants (Cao et al., 2021). The application of rhizosphere microorganisms to promote plant growth has been widely carried out worldwide (Goswami et al., 2019; Aloo et al., 2021; Oliveira et al., 2022).

#### 3.2.8. Keywords with citation burst

**Figure 5:**
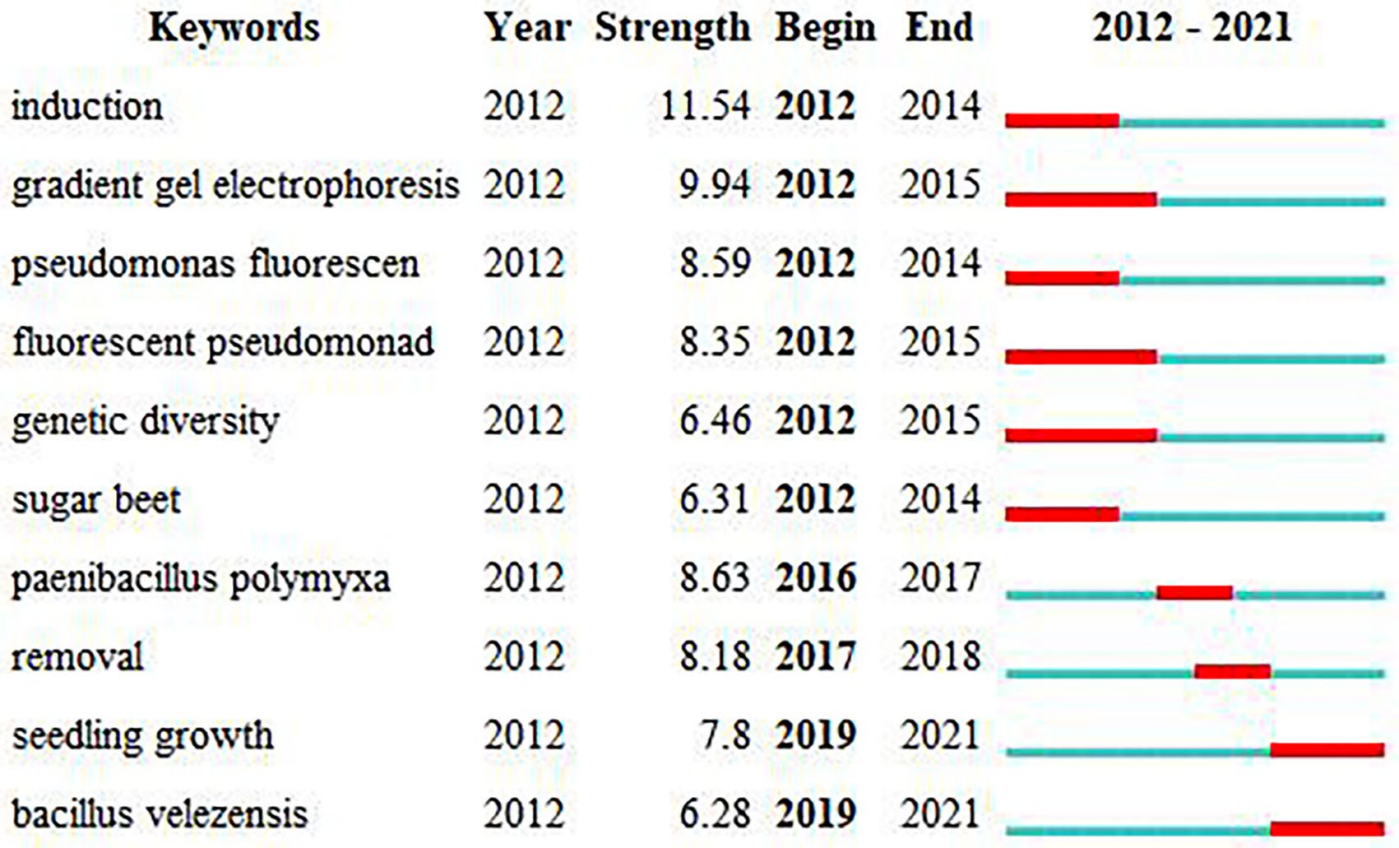
Top 10 keywords with the strongest citation bursts.

We used burst detection to identify articles that attracted the attention of peer researchers during the study period (Liu et al., 2022). The top keywords with the most robust citation burst were shown in Figure 5. There were 51 keywords with the top burst strength among the total keywords. We were particularly interested in those keywords with research significance, which indicated the evolving trend of rhizosphere microorganisms (Figure 5). During the study period from 2012 to 2021, induction had the highest burst strength, followed by gradient gel electrophoresis, *Paenibacillus polymyxa*, and *Pseudomonas fluorescen*. Simultaneously, we can see that seeding growth and bacillus velezensis became the research focus after 2019, and may also be the research hot spots in the future period.

#### 3.2.9. Thematic evolution map of keywords in articles

**Figure 6:**
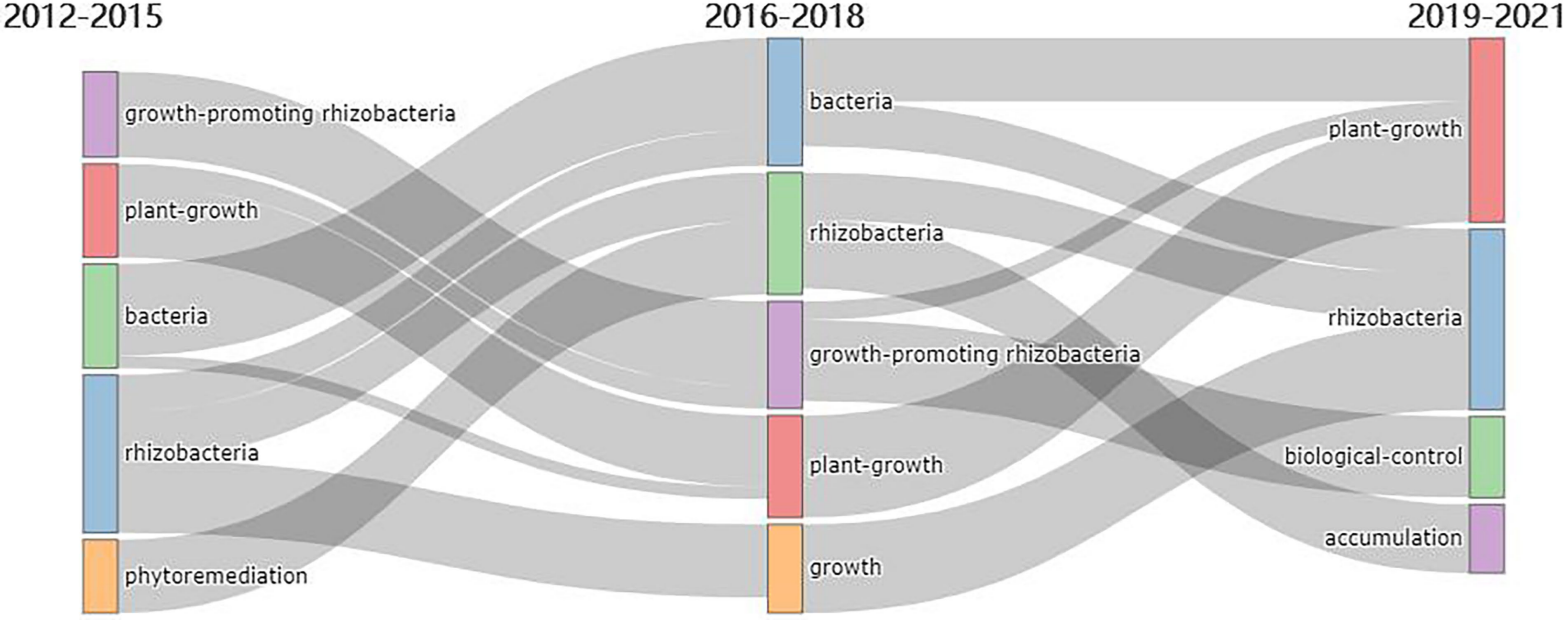
Thematic evolution map of publications in rhizosphere microorganism from 2012 to 2021.

The thematic evolution of keywords plus during the last decade showed a clear shift in scientific fields (Liu et al., 2019). Phytoremediation-related keywords disappeared after 2015 (Figure 6). Phytoremediation technology referred to an environmental pollution control technology that absorbs, degrades, volatilizes and concentrates pollutants through the plant and microbial systems based on plant tolerance, decomposition ability or the super-accumulation ability of some chemical elements (Wang et al., 2015). Phytoremediation was one of the main means of environmental treatment of heavy metal pollution (Jacquiod et al., 2022; Raklami et al., 2022). Compared with traditional physicochemical remediation methods, phytoremediation had the characteristics of low technical cost, simple process and environmental friendliness. In recent years, phytoremediation has been widely used in mine reclamation and remediation of heavy metal contaminated sites (Ali et al., 2013; Mahar et al., 2016). This approach is important in the current scenario of a circular economy (Benidire et al., 2021).

In addition, plant growth and rhizobacteria were found to be perpetual areas of interest. Plant roots change the physicochemical properties of soil through rhizosphere secretions, such as soil pH, soil aggregate structure, available nutrients, available oxygen, the concentration of bacteriostatic substances, and mimicry of the quorum sensing system, thus affecting rhizosphere microbial community components (Biedrzycki and Bais, 2013). Rhizosphere microorganisms and plant roots form a special ecosystem, which played an important role in the decomposition of soil organic matter around plant roots, and the fixation, transformation and absorption of nutrients. Underground microbial communities in specific ecosystems play an important role in plant productivity, plant community composition and diversity (Bi et al., 2022; Lv et al., 2022).

Bio-control was a new thematic plus in recent 3 years. Research showed that bio-control microorganisms can promote growth (Chopra et al., 2020; Sherpa et al., 2021), improve the cold resistance of crops (Theocharis et al., 2012), drought resistance (Mehmood et al., 2021), and heavy metal resistance (Rajkumar et al., 2012). Therefore, we can predict that bio-control will become a hot research topic in the field of rhizosphere microorganisms in the future (Win et al., 2021).

## 4. Conclusion

In this paper, CiteSpace 6.1.3 and R 5.3.1 were used to conduct quantitative statistics and visual analysis of the literature information related to rhizosphere microorganisms from WoSCC from 2012 to 2021. It showed the annual change, knowledge base, research hotspots, and trends, distribution of core publishing countries, research institutions, and authors in the field of rhizosphere microorganism research. The following conclusions were drawn:

• The amount of research articles on rhizosphere microorganisms has been on the rise in the past decade.

• The top three countries in terms of the number of articles issued were China, India and Pakistan, while the United States and Germany were the countries with the highest number of co-published papers with other countries.

• The core research content in this field were bio-control, bacterial community, ACC deaminase, phytoremediation, induced systematic resistance, and plant growth promotion.

• Seeding growth, bacillus velezensis, plant growth, and bio-control are currently and may be the hot spots in the field of rhizosphere microorganism research for a long time in the future.

## Author contributions

SS, RX, and ML were performed material preparation, data collection, and analysis. SS written first draft of the manuscript. WZ and LW contributed to the conception, design and supervision of this study. All authors commented on the previous versions of the manuscript, read and approved the final manuscript.

## Funding

This research was funded by the National Key Research and Development Program of China (2022YFC2601305).

## Conflict of interest

The authors declare that the research was conducted in the absence of any commercial or financial relationships that could be construed as a potential conflict of interest.

## Publisher’s note

All claims expressed in this article are solely those of the authors and do not necessarily represent those of their affiliated organizations, or those of the publisher, the editors and the reviewers. Any product that may be evaluated in this article, or claim that may be made by its manufacturer, is not guaranteed or endorsed by the publisher.
